# Resolving the complex *Bordetella pertussis* genome using barcoded nanopore sequencing

**DOI:** 10.1099/mgen.0.000234

**Published:** 2018-11-21

**Authors:** Natalie Ring, Jonathan S. Abrahams, Miten Jain, Hugh Olsen, Andrew Preston, Stefan Bagby

**Affiliations:** ^1^​ Department of Biology and Biochemistry and the Milner Centre for Evolution, University of Bath, Bath, UK; ^2^​ UC Santa Cruz Genomics Institute, 1156 High Street, Santa Cruz, CA 95064, USA

**Keywords:** genome assembly, Oxford nanopore, long-read sequencing, *Bordetella pertussis*, benchmarking, duplications

## Abstract

The genome of *Bordetella pertussis* is complex, with high G+C content and many repeats, each longer than 1000 bp. Long-read sequencing offers the opportunity to produce single-contig *B. pertussis* assemblies using sequencing reads which are longer than the repetitive sections, with the potential to reveal genomic features which were previously unobservable in multi-contig assemblies produced by short-read sequencing alone. We used an R9.4 MinION flow cell and barcoding to sequence five *B. pertussis* strains in a single sequencing run. We then trialled combinations of the many nanopore user community-built long-read analysis tools to establish the current optimal assembly pipeline for *B. pertussis* genome sequences. This pipeline produced closed genome sequences for four strains, allowing visualization of inter-strain genomic rearrangement. Read mapping to the Tohama I reference genome suggests that the remaining strain contains an ultra-long duplicated region (almost 200 kbp), which was not resolved by our pipeline; further investigation also revealed that a second strain that was seemingly resolved by our pipeline may contain an even longer duplication, albeit in a small subset of cells. We have therefore demonstrated the ability to resolve the structure of several *B. pertussis* strains per single barcoded nanopore flow cell, but the genomes with highest complexity (e.g. very large duplicated regions) remain only partially resolved using the standard library preparation and will require an alternative library preparation method. For full strain characterization, we recommend hybrid assembly of long and short reads together; for comparison of genome arrangement, assembly using long reads alone is sufficient.

## Data Summary

1. Final sequence read files (fastq) for all five strains have been deposited in the Sequence Read Archive, BioProject PRJNA478201, accession numbers SAMN09500966, SAMN09500967, SAMN09500968, SAMN09500969, SAMN09500970.

2. A full list of accession numbers for Illumina sequence reads is available in Table S1 (available in the online version of this article).

3. Assembly tests, basecalled read sets and reference materials are available from figshare: https://figshare.com/projects/Resolving_the_complex_Bordetella_pertussis_genome_using_barcoded_nanopore_sequencing/31313.

4. Genome sequences for *B. pertussis* strains UK36, UK38, UK39, UK48 and UK76 have been deposited in GenBank, accession numbers: CP031289, CP031112, CP031113, QRAX00000000, CP031114.

5. Source code and full commands used are available from Github: https://github.com/nataliering/Resolving-the-complex-Bordetella-pertussis-genome-using-barcoded-nanopore-sequencing.

**Impact Statement**

Over the past two decades, whole genome sequencing has allowed us to understand microbial pathogenicity and evolution to an unprecedented degree. However, repetitive regions, like those found throughout the *Bordetella pertussis* genome, have confounded our ability to resolve complex genomes using short-read sequencing technologies alone. We have used nanopore sequencing, which can generate reads longer than these problematic repetitive regions, to resolve multiple *B. pertussis* genomes with a single flow cell. The resolved genomes can be used to visualize previously predicted genome rearrangements and, in addition, the inability of our long reads to resolve some of our genomes has allowed us to infer the presence of previously unidentified ultra-long duplications in two of our five strains. Thus, our findings point towards unanticipated genome-level genetic variation in strains which appear otherwise monomorphic at the nucleotide level. This work expands the recently emergent theme that even the most complex genomes can be resolved with sufficiently long sequencing reads. Our optimization process, moreover, shows that the analysis tools currently favoured by the sequencing community do not necessarily produce the most accurate assemblies for all organisms; pipeline optimization may therefore be beneficial in studies of unusually complex genomes.

## Introduction

*Bordetella pertussis* is the pathogenic bacterium which causes most cases of whooping cough (pertussis). Pertussis was a major medical burden prior to the international introduction of vaccination in the 1940s and 1950s. Widespread vaccine uptake greatly reduced incidence of the disease in developed countries. Original whole-cell vaccines were replaced by new acellular vaccines throughout the 1990s and early 2000s. The acellular vaccines contain one to five of the *B. pertussis* protein antigens pertactin (Prn), pertussis toxin (Pt), filamentous haemagglutinin (FHA), and the fimbrial proteins Fim2 and Fim3. Despite continued high levels of pertussis vaccination coverage, since the early 1990s the number of cases of whooping cough has increased in many countries [1, 2].

Suggested causes for this resurgence include improved diagnostic tests and awareness, waning immunity as a result of the switch to acellular vaccination, and genetic divergence of circulating *B. pertussis* from the vaccine strains due to vaccination-induced selection pressure [3–5]. A global survey of strains from the pre-vaccine, whole-cell vaccine and acellular vaccine eras showed that the genome of *B. pertussis*, widely regarded as a monomorphic and slowly evolving organism, has been evolving since the introduction of the whole-cell vaccine [6]. Analysis of strains from several recent epidemics showed the rate of evolution of the genes encoding vaccine antigens has increased since the switch to the acellular vaccine [7–10].

The *B. pertussis* genome contains up to 300 copies of a 1053 bp insertion sequence (IS), IS*481*. A smaller number of copies of IS*1002* (1040 bp) and IS*1663* (1014 bp) contribute further complexity to the genome. These regions of repetition mean that assembly of closed, single-contig *B. pertussis* genomes using short-read sequencing, which produces reads shorter than the IS repeats, has been particularly difficult: most genome sequences available on NCBI comprise several hundred contigs, or at least one contig per IS copy. Over the last decade, many studies have shown that reads longer than the longest repeat are required to resolve regions of high complexity [11–18]. Assembly of closed genomes may reveal genomic features which were previously unobservable in multi-contig assemblies; this is particularly true for genomes which contain a high number of IS copies, as insertion sequences are known to impact genomic structure via rearrangement, deletion and, more rarely, duplication [19, 20].

In 2016, Bowden *et al.* [21] were the first to use long reads, together with Illumina short reads, to conduct a survey of *B. pertussis* strains which had circulated during two whooping cough epidemics, in the USA, in 2010 and 2012. Assembling closed genomes for these epidemic isolates revealed extensive genomic arrangement differences between isolates which appeared to be otherwise closely related. Bowden *et al.* concluded that further comprehensive whole genome studies are required to fully understand the international resurgence of whooping cough. More recently, Weigand *et al.* showed that the *B. pertussis* genome continues to undergo structural rearrangement on a frequent basis, usually mediated by IS*481* [22]. As well as causing structural rearrangement, IS elements have also repeatedly been shown to be responsible for the ongoing reduction of the *B. pertussis* genome via gene deletion [23–26].

Bowden *et al.* and Weigand *et al.* both used Pacific Biosciences (PacBio) long read sequencing, which has high start-up costs, and lacks the portability needed for on-the-ground epidemic surveillance. In contrast, Oxford Nanopore Technology (ONT)’s MinION nanopore sequencer has relatively low start-up costs. Recent improvements to flow cell yield and the introduction of barcoded library preparation make per-sample MinION costs comparable to those of PacBio or Illumina [15, 27, 28]. In addition, the pocket-sized MinION sequencer is portable, enabling in-the-field sequencing [29–31].

Here we test the ability of barcoded nanopore sequencing, together with a variety of available data analysis tools, to resolve the genomes of five *B. pertussis* strains from a UK epidemic, which were previously unclosed and comprised many contigs assembled using short reads sequenced with Illumina’s MiSeq [7]. We then briefly investigate the resulting genomes to identify any previously unobserved features, with a particular focus on the genome of one strain which remained unresolved by our hybrid assembly strategy.

## Methods

Full method and bioinformatics procedures are described at: https://github.com/nataliering/Resolving-the-complex-Bordetella-pertussis-genome-using-barcoded-nanopore-sequencing.

All data analysis was carried out using the Medical Research Council’s Cloud Infrastructure for Microbial Bioinformatics (CLIMB) [32].

### Strain isolation and Illumina sequencing

Five strains originally isolated during the UK 2012 whooping cough epidemic were obtained from the National Reference Laboratory, Respiratory and Vaccine Preventable Bacteria Reference Unit, at Public Health England. Short-read sequencing data were generated previously, using genomic DNA (gDNA) extracted using a DNeasy Blood and Tissue kit (Qiagen), multiplex library preparation and Illumina sequencing [7]. Full details, including accession numbers, are included in Table S1.

### DNA extraction

Strains were stored at −80 °C in PBS/20 % glycerol at the University of Bath. They were grown on charcoal agar plates (Oxoid) for 72 h at 37 °C. All cells were harvested from each plate and resuspended in 3 ml PBS. The optical density of each cell suspension was measured at 600 nm, and volumes of suspension equating to an OD of 1.0 (~2×10^9^ *B. pertussis* cells) in 180 µl were pelleted in a microcentrifuge for 2 min at 12 000 ***g***. gDNA was extracted from each pellet using a GenElute bacterial genomic DNA kit (Sigma Aldrich) according to the manufacturer’s instructions, including the optional RNAase A step and a two-step elution into 200 µl elution buffer (10 mM Tris-HCl, 0.5 mM EDTA, pH 9.0). QuBit fluorometry (dsDNA HS kit; Invitrogen) was used to measure gDNA concentration, and Nanodrop spectrometry (ThermoFisher Scientific) was used to assess gDNA purity.

### Nanopore library preparation and MinION sequencing

In total, 1.5 µg of gDNA per strain was concentrated using a 2.5× SPRI clean-up (AMPure XP beads; Beckman Coulter), eluting into 50 µl of nuclease-free (NF) water (Ambion). Then, 48 µl of this was sheared to 20 kb using g-tubes (Covaris), according to the manufacturer’s instructions.

Sequencing libraries were prepared for all samples using ONT's 1D ligation sequencing kit (SQK-LSK108) with native barcoding (EXP-NBD103), according to the manufacturer’s instructions. Ten barcodes were used, two per strain (see Table S2 for full details). After library preparation, different volumes of samples were combined to produce an equi-mass pool for eight samples; two samples had much lower concentration after library preparation so were pooled in full. A total mass of 712.5 ng was pooled in 208.9 µl NF water, which was concentrated to 50 µl by 2.5× SPRI clean-up. Full details of mass pooled per sample are given in Table S2. This pooled library (712.5 ng in 50 µl) was used for sequencing adapter ligation.

The final sequencing library was loaded onto an R9.4 flow cell and sequenced for 48 h using a MinION MK1b device with MinKNOW sequencing software (protocol NC_48h_Sequencing_Run_FLOMIN106_SQK-LSK108).

### Additional basecalling and demultiplexing

The fast5 files were basecalled using ONT’s Albacore (v2.1.3) program, with barcode binning. As suggested by Wick *et al.* [15], Porechop was then used to demultiplex the Albacore reads, keeping only those for which Albacore and Porechop agreed on the bin. The Albacore+Porechop fastq files were deposited in the Sequence Read Archive (SRA) with accession codes SAMN09500966 to SAMN0900970. Full details of all read sets (including reads output by MinKNOW’s concurrent basecalling algorithm) are given in Table S1.

### Assembly of short-read-only drafts

Assuming the available Illumina data to have typically low error, short-read-only genome sequences were assembled for each strain using ABySS (v2.0.3) [33]. Prior to assembly, reads were prepared using Trimmomatic (v0.34) [34], which trimmed the first 10 bases of each read, and discarded any reads whose four-base sliding-window q-score fell below 32. These assemblies had low contiguity, but theoretically high accuracy.

### Comparison of raw reads

A shell script, ‘summary_stats’, was used to give the total number of reads, mass sequenced and minimum, maximum, mean and median read lengths for each set of raw reads. Summary_stats uses seq_length.py [35] and all_stats. All are available from https://github.com/nataliering/Resolving-the-complex-Bordetella-pertussis-genome-using-barcoded-nanopore-sequencing.

Raw percentage identity was estimated by comparing each read set to the *B. pertussis* reference genome (Tohama I, NC_002929.2). As the UK 2012 strains were not expected to be identical to the Tohama I sequence, read error was also estimated by comparison with the respective Illumina-only assemblies. The comparison was conducted using BWA MEM [36] and samtools stats [37], which produces a long output file including ‘error rate’ [% identity was calculated from this: 100 − (error rate*100)]. Raw_error (https://github.com/nataliering/Resolving-the-complex-Bordetella-pertussis-genome-using-barcoded-nanopore-sequencing/blob/master/raw_error) produces a stats file using this method, given a read set and reference genome. Using the same BWA MEM output, raw read coverage of the Tohama I reference genome was checked using samtools depth and visualization with a rolling window in R.

Finally, raw G+C content was calculated using GC_calculator, which outputs the percentage G+C content of a given fasta file (https://github.com/nataliering/Resolving-the-complex-Bordetella-pertussis-genome-using-barcoded-nanopore-sequencing/blob/master/GC_calculator).

### Assembly tool testing – nanopore only

The Albacore+Porechop reads for one strain, UK36, were used to test a variety of *de novo* assembly strategies. Four community-built assembly tools were trialled: ABruijn (now called Flye, v1.0 and v2.3.2 respectively), Canu (v1.5), Miniasm with Minimap/Minimap2 (v0.2-r128, v0.2-r123 and v2.0-r299-dirty, respectively) and Unicycler (v0.4.4) [38–41].

Canu has a standalone option to conduct pre-assembly read correction. This was used to correct the 359× coverage UK36 read set to 40× coverage of more accurate reads. Each assembly tool was then trialled with and without pre-assembly read correction. As Canu’s read correction step is relatively time-consuming as regards CPU, an alternative was also trialled. Filtlong (https://github.com/rrwick/Filtlong) does not correct reads, but produces read sets comprising the longest and most accurate reads, up to a given level of coverage; 40× and 100× coverage were trialled here.

Finally, Racon (v.1.2.0) [42] was tested to determine whether the draft assemblies could be improved by post-assembly polishing. After each Racon polish, the accuracy of the assembly produced was estimated. If an improvement was observed, another round of polishing was conducted, up to a total of five rounds. Once two successive rounds of polishing showed no further improvement, no further Racon polishes were conducted. For Unicycler, no manual Racon polishes were conducted, because Racon polishing is part of the Unicycler assembly process. After Racon polishing, each assembly was further polished with a single round of Nanopolish (v0.9.0) [14].

Testing exhaustive combinations of each of these steps produced 28 draft assemblies for each of the four assembly tools tested (ABruijn/Flye, Canu, Miniasm and Unicycler), a total of 112 draft UK36 assemblies (see Table S3 for all combinations).

### Assembly tool testing – hybrid

As Illumina reads were already available for the strains sequenced here, a variety of hybrid *de novo* assembly strategies were also tested. Using Pilon (v1.22) [43], the best nanopore-only assembly for each of the assembly tools was polished with the Illumina reads, up to a total of five rounds. In addition, a hybrid assembly was produced using Unicycler’s hybrid mode, which both combines read sets for assembly, and conducts several rounds of Racon and Pilon polishes automatically. Finally, the hybrid assembly mode of SPAdes (v3.12.0) [44] was tested. These hybrid tests produced another 22 draft assemblies (Table S4).

### Assessing assembly accuracy

Summary_stats was used to determine the number of contigs, and contig length for each draft assembly. The percentage identity of each draft compared to the Illumina-only draft was estimated using a method developed by Wick *et al.* [45]. Their chop_up_assembly.py and read_length_identity.py scripts were used to generate percentage identity values for 10 kbp sections along the entirety of each assembly, and a custom shell script, assembly_identity (https://github.com/nataliering/Resolving-the-complex-Bordetella-pertussis-genome-using-barcoded-nanopore-sequencing/blob/master/assembly_identity), was used to calculate the mean percentage identity of the whole.

Quality metrics for each assembly were produced using Quast (v4.5) [46] and BUSCO (v1.22) [47]. In addition, a method developed by Watson [48], Ideel (https://github.com/mw55309/ideel), was used to assess the effect of any erroneous indels in the final UK36 hybrid assembly.

### Comparing genome arrangement

After the best nanopore-only and hybrid assembly pipelines were identified for UK36, the pipelines were used to produce draft assemblies for the remaining four strains. The hybrid assembly for each strain was annotated with Prokka (v1.12) [49] using the proteins from Tohama I as a reference. The genomes were also submitted to GenBank (accession numbers CP031289, CP031112, CP031113, QRAX00000000 and CP031114).

The arrangement of each nanopore-only assembly was compared to that of each hybrid using progressiveMauve (v20150226 build 10) [50]. Finally, the nanopore-only assemblies for each strain were compared to each other, also using progressiveMauve. Prior to these alignments, each draft was manually rearranged so that the first gene after the *B. pertussis* origin of replication, *gidA*, was at the beginning of the sequence. gidA_blast (https://github.com/nataliering/Resolving-the-complex-Bordetella-pertussis-genome-using-barcoded-nanopore-sequencing/blob/master/gidA_blast) locates the *gidA* sequence in the draft to enable manual rearrangement. Later, this same process was used to identify IS element copies in the assembled genomes. If a tool assembled the complementary strand instead of the template (as identified by the results of gidA_blast), a reverse complement of the draft sequence was generated using reverse_complement (https://github.com/nataliering/Resolving-the-complex-Bordetella-pertussis-genome-using-barcoded-nanopore-sequencing/blob/master/reverse_complement).

## Results

### Sequencing yield

**Fig. 1.**
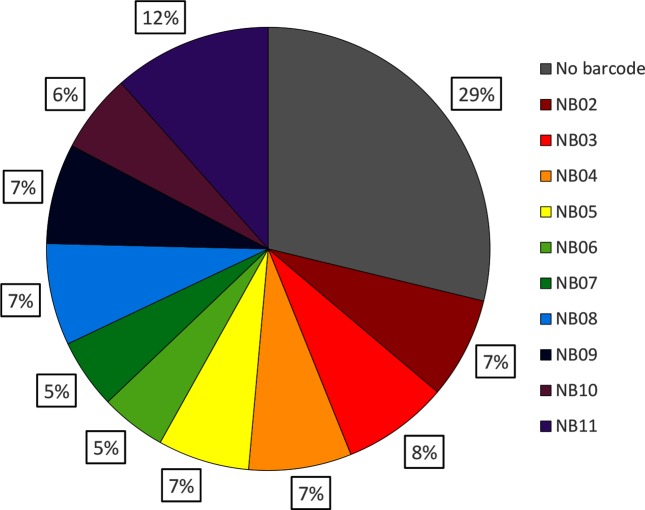
The Albacore+Porechop reads were used to assess barcode distribution. This showed that a large portion of the raw reads was placed into the ‘no barcode’ bin, meaning Albacore and Porechop either did not agree on a barcode, or no recognizable barcode was present. Otherwise, the barcodes were largely well distributed.

During the 48 h MinION sequencing run, 1 803 648 reads were generated, equating to 9.73 Gbp of sequence. In total, 28.78 % of these reads (574 053 reads, 2.8  Gbp) were not assigned to the correct barcode bin during demultiplexing, leaving 6.93 Gbp (1 229 595 reads) of useable sequencing data (Fig. 1). Normalized yield per barcode (taking into account nanograms of gDNA included in the pooled sequencing library) was particularly high for one barcode (NB11, 15.28 Mbp ng^−1^) but otherwise relatively consistent, ranging from 7.38 to 10.28 Mbp ng^−1^ with a mean yield of 9.06 Mbp ng^−1^ (se=0.37). Mean read length for the full read set was 5689 bp. Read lengths ranged from 4 to 201 977 bp.

The Albacore-demultiplexed reads were re-demultiplexed using Porechop, which keeps only the reads for which both tools agree on the barcode identified. This additional step resulted in a small but significant improvement in identity compared to Illumina-only assembly: 82.43 to 82.52 % (*n*=5, paired *t*-test *P*<0.001). Consequently, the reads used for data pipeline testing were those that had been basecalled and demultiplexed by Albacore, followed by Porechop re-demultiplexing.

For full results, including which barcode was assigned to each sample, see Table S1.

### Assembly tool testing – nanopore-only

Table 1 shows the quality measurements for the best nanopore-only assembly per tool trialled. All tools tested were able to resolve the nanopore long reads for UK36 into a complete, closed contig, using default assembly options with no manual intervention. In total, 112 different tool combinations were trialled. Alignment of drafts from different tools using progressiveMauve revealed that each tool also assembled the genome into the same arrangement. However, the length of the draft assemblies showed some variation: 3.984 to 4.134 Mbp, with a mean length of 4.108 Mbp.

**Table 1.** Best *de novo* assembly options and quality measurements for nanopore-only assemblies

**Assembler**	**Pre-assembly read correction**	**Pre-assembly read filtering (× coverage)**	**Rounds of Racon polishing**	**Polishing with Nanopolish**	**Contigs**	**Assembly length (Mbp)**	**Percentage identity compared to Illumina-only**	**BUSCOs present/fragment/missing (of 40)**
ABruijn	Yes	No	0	Yes	1	4.105	99.59	37/2/1
Canu	Yes	No	4	Yes	1	4.133	99.54	36/1/3
Flye	Yes	No	0	Yes	1	4.108	99.56	35/3/2
Miniasm+Minimap	Yes	No	5	Yes	1	4.111	99.55	37/0/3
Unicycler	Yes	No	8*	Yes	1	4.107	99.55	35/2/3

*The rounds of Racon listed for Unicycler were carried out as part of the Unicycler protocol; no manual rounds of Racon were conducted.

Comparing like-for-like assemblies before and after polishing shows that Nanopolish improves identity by 0.216 % on average (*n*=16, paired *t*-test *P*<0.001). Polishing with Racon produced inconsistent results: identity decreased after Racon polishing of ABruijn and Flye drafts, increased by 2.01 % after the optimal number of polishing rounds for pre-corrected non-ABruijn/Flye drafts (*n*=3), and increased by 15.15 % after optimal rounds for non-ABruijn/Flye drafts with no pre-correction (*n*=4). The mean number of Racon polishes required to reach optimal percentage identity (after which it began to decrease) was 4.75 (*n*=7).

The assembly with greatest percentage identity compared to the Illumina-only draft (99.59 %) combined pre-assembly read correction with Canu, assembly with ABruijn and post-assembly polishing with Nanopolish. The assemblies were also assessed using BUSCO, which searches draft assemblies for copies of benchmarking universal single-copy orthologues (BUSCOs). BUSCOs are sets of core genes which are likely to appear universally in related organisms. A set of 40 such core genes from the *Escherichia coli* genome are used as the gram-negative bacterial BUSCOs; if a genome has been assembled accurately, the tool BUSCO is more likely to be able to identify these 40 genes within its sequence. Of the drafts assessed here, the ABruijn assembly contained the highest number of identifiable BUSCOs (37 full and two partial, of the full set of 40; see Table 1 for full results).

### Assembly tool testing – hybrid

A number of hybrid assembly strategies were trialled, including polishing a long-read assembly with short reads, scaffolding short-read contigs with long reads, and using both short and long reads together during assembly (Table 2 shows the best draft produced by each tool). Scaffolding short-read contigs with long reads using SPAdes produced one of the highest accuracy assemblies (99.68 %), but did not fully resolve the genome, as six contigs remained. No further polishing was attempted with this SPAdes assembly, as polishing would not close the remaining gaps between the contigs.

**Table 2.** Best *de novo* assembly options and quality measurements for hybrid assemblies

**Assembler**	**Pre-assembly read correction**	**Pre-assembly read filtering (× coverage)**	**Assembly includes short reads?**	**Rounds of Racon polishing**	**Polishing with Nanopolish**	**Rounds of Pilon polishing**	**Contigs**	**Assembly length (Mbp)**	**Percentage identity compared to Illumina-only**	**BUSCOs present/fragment/missing (of 40)**
ABruijn	Yes	No	No	0	Yes	3	1	4.109	99.67	40/0/0
Canu	Yes	No	No	4	Yes	3	1	4.130	99.66	40/0/0
Flye	Yes	No	No	0	Yes	3	1	4.108	99.67	40/0/0
Miniasm+Minimap	No	No	No	5	Yes	4	1	4.107	99.66	40/0/0
SPAdes	Yes	No	Yes	n/a	n/a	n/a	6	4.105	99.68	40/0/0
Unicycler	Yes	No	Yes	4*	No	8*	1	4.107	99.68	40/0/0

*The rounds of Racon and Pilon listed for Unicycler were carried out as part of the Unicycler protocol; no manual rounds of polishing were conducted for this assembly.

The best hybrid assemblies per tool were significantly more accurate than the best nanopore-only assemblies per tool, with a mean identity improvement of 0.11 % (hybrid *n*=6, nanopore-only *n*=5, paired *t*-test *P*<0.001). In addition, all hybrids contained all 40 identifiable BUSCOs, and all except the SPAdes hybrid were single closed contigs and showed the same arrangement when aligned using progressiveMauve.

The best single-contig hybrid assembly, with 99.68 % identity, was produced using Unicycler’s hybrid option, which uses SPAdes, Minimap, Miniasm, Racon and Pilon. Table S5 shows the results from all nanopore-only and hybrid tests.

### Assembly and annotation of all strains

**Fig. 2.**
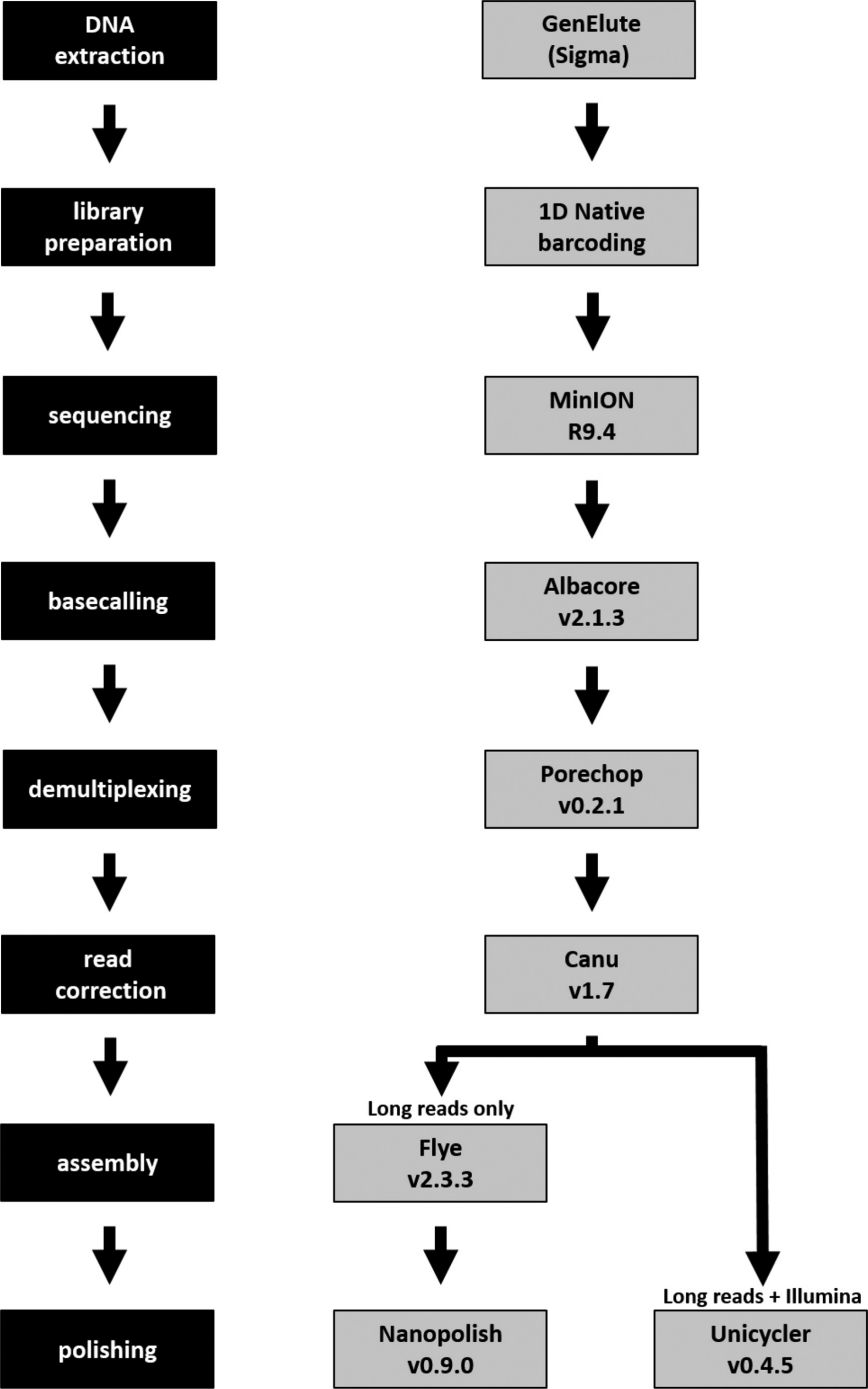
Our nanopore-only and hybrid sequencing pipelines, developed through extensive testing of available tools.

Using the nanopore-only and hybrid pipelines defined through the tests described here (Fig. 2), draft genomes were assembled for all five UK strains sequenced during our barcoded run. The assemblies were assessed for percentage identity compared to each strain’s Illumina-only assembly, G+C content, genome length and number of key IS element features; they were also annotated using Prokka. The full results of this analysis are shown in Tables 3, S6 and S7.

**Table 3.** Assembly statistics for five UK *B. pertussis* strains, assembled using our hybrid pipeline

**Pipeline**	**Strain**	**Contigs**	**Genome length (Mbp)**	**G+C content (%)**	**Percentage identity compared to Illumina-only**	**No. of genes predicted**	**IS*481* copies**	**IS*1002* copies**	**IS*1663* copies**
Nanopore-only	UK36	1	4.108	67.69	99.47	4698	258	8	17
	UK38	1	4.108	67.69	99.49	4741	258	8	17
	UK39	1	4.109	67.70	99.48	4588	258	8	17
	UK48	1	4.114	67.70	99.47	4610	262	8	17
	UK76	1	4.113	67.70	99.32	4608	262	8	17
Hybrid	UK36	1	4.107	67.70	99.68	3757	258	8	17
	UK38	1	4.108	67.70	99.69	3757	258	8	17
	UK39	1	4.108	67.70	99.69	3804	258	8	17
	UK48	2	4.112	67.70	99.68	3763	262	8	17
	UK76	1	4.113	67.70	99.54	3753	262	8	17

The hybrid assembly for one strain, UK76, had slightly lower percentage identity (99.54 %) than the other strains, each compared to their respective Illumina-only ABySS assembly. Discounting UK76, the assemblies had a mean identity of 99.69 % (*n*=4). The G+C content of the strains varied little: the content for all strains was 67.70 % when rounded to two decimal places. The number of genes predicted by Prokka was also relatively consistent, varying from 3757 to 3804.

The UK36 proteins predicted by Prokka were assessed by Ideel, which searched the Trembl database [51] for similar proteins. The length of the Prokka-predicted proteins was divided by those of the identified similar Trembl proteins; a perfect match would equal 1.0. This method, therefore, indicates whether indels in a draft sequence cause frameshifts which subsequently lead to truncated (or over-long) protein prediction. After manual curation to remove results which represented genes known to be fully present in other *Bordetella* species but truncated in *B. pertussis*, over 98 % of Prokka-predicted genes had a Prokka:Trembl length ratio of greater than 0.9. This suggests that the residual error in the hybrid assemblies does not cause substantial annotation problems, so the hybrid assemblies for all five strains were submitted to GenBank (accession numbers CP031289, CP031112, CP031113, QRAX00000000 and CP031114).

### Comparison of genomic structure of all strains

**Fig. 3.**
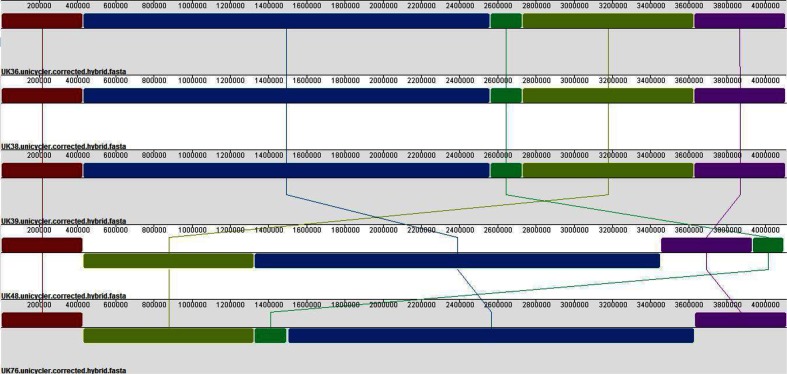
Alignment of our five sequenced strains, showing genomic rearrangement. Our five UK *B. pertussis* strains (UK36, UK38, UK39, UK48 and UK76) were assembled using our nanopore-only pipeline, resulting in single, closed-contig, assemblies. The closed assemblies were aligned with progressiveMauve, which showed that even strains which are closely temporally related can display different genomic arrangements.

All strains were assembled into single contigs using the nanopore-only pipeline. These assemblies were aligned using progressiveMauve (Fig. 3), displaying genomic rearrangement between strains; three, UK36, UK38 and UK39, shared exactly the same arrangement, whilst UK48 and UK76 were rearranged.

**Fig. 4.**
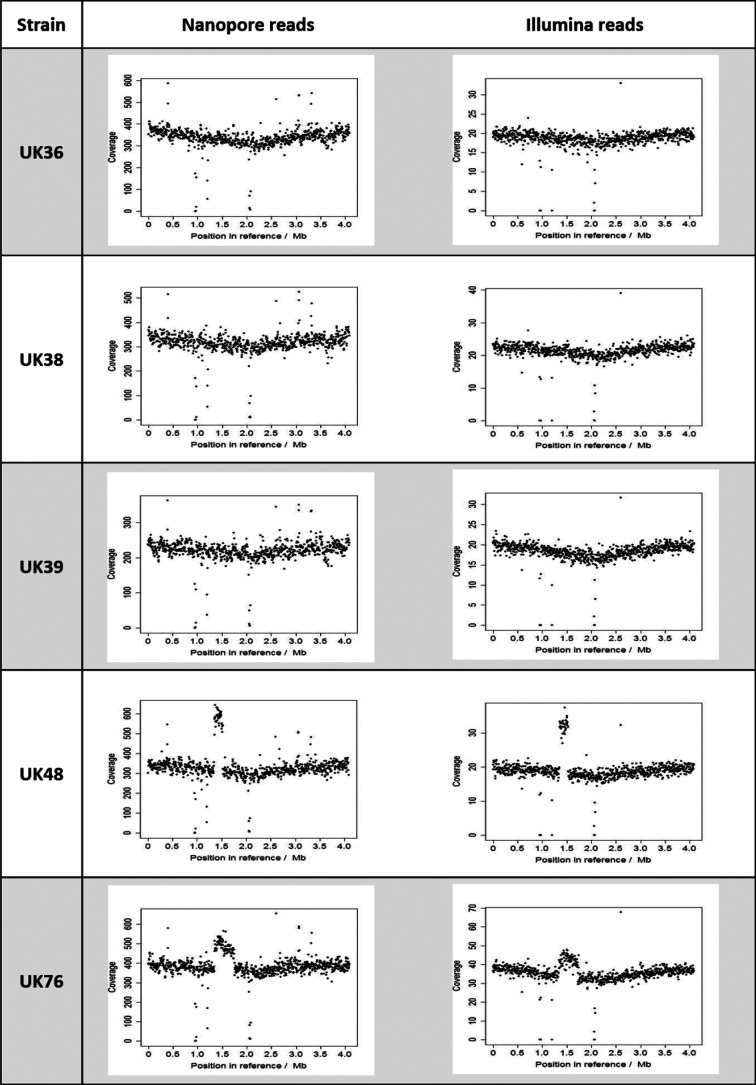
Alignment of nanopore reads to the Tohama I reference sequence compared to alignment of Illumina reads to the Tohama I reference sequence. Raw reads from each sequencer were aligned to the reference using BWA MEM, followed by coverage calculation with samtools depth. The coverage of three strains (UK36, UK38 and UK39) was consistent across the whole reference genome, whereas UK48 and UK76 coverage was enriched in certain locations. In UK48, a large section from 1.35 to 1.53 Mbp into the reference appears to have exactly twice as much coverage as the rest of the genome. In UK76, a section from 1.38 to 1.68 Mbp is enriched by 25%. The coverage abnormalities seen in UK48 and UK76 are present in both sets of reads, suggesting they are not the result of a quirk in sequencing method, or contamination.

Of the hybrid assemblies, two strains, UK48 and UK76, had longer genomes than the others (4.112 and 4.113 Mbp, respectively, compared to 4.108 Mbp), which corresponds with them also having more copies of the most abundant IS element, IS*481*. All strains but one were assembled into single contigs. The remaining strain, UK48, was assembled into five contigs (N50=3.934 Mbp). Of these, three were shorter than 500 bp, and were subsequently discarded. The remaining two contigs were 3 934 355 and 178 023 bp. Mapping the raw UK48 reads to the Tohama I reference sequence revealed a section of almost 200 kbp, located between 1.35 and 1.53 Mbp, which had double the read depth of the rest of the reference; the doubled read depth suggests that this section of the genome is duplicated in UK48. No other strain had a similarly duplicated section, although the coverage of UK76 was enriched by around 25 % at the same locus (Fig. 4), potentially indicating a heterogeneous UK76 population, of which a subset (i.e. 25 %) of cells carries a duplication. These abnormalities are also present in the Illumina reads, which were obtained approximately 5 years before our nanopore reads (Fig. 4), but had not been identified previously using the short reads alone.

## Discussion

### Are residual unresolved ultra-long repeats present in some strains?

Our primary aim in this study was to determine whether long reads produced by nanopore sequencing using ONT’s MinION can be used to produce closed *B. pertussis* genome sequences, which will enable visualization of large-scale inter-strain genomic differences, and may further reveal previously hidden genomic features. Our nanopore-only assembly pipeline produced closed-contig assemblies for all five strains sequenced here, allowing visualization and validation of previously predicted IS-mediated genomic rearrangements. In addition, the inability of our long reads to produce a closed hybrid assembly for UK48 has revealed a separate, unpredicted, genomic feature in our UK strains.

The region of enriched coverage between 1.35 and 1.53 Mbp in the Tohama I reference genome observed in the UK48 reads (Fig. 3) is likely to indicate a large (almost 200 kbp) duplication of that region which is present in UK48 but not in the reference. A less obvious duplication may also be present in the genome of UK76: a 300 kbp region from 1.38 to 1.68 Mbp shows 125 % coverage. The presence of the same abnormalities in other read sets for both strains suggests that they have not been caused by contamination (Fig. 3). Similar duplicated regions have been observed previously in a very small number of French and Finnish strains (fewer than five) through microarray-based studies in 2006 and 2007 [25, 26]. More recently, Weigand *et al*. [52, 53] noted complex duplications in two US strains and two Indian vaccine-reference strains; these genomes were long-read sequenced with PacBio, but resolution of the duplications was only possible with optical mapping. The locus found to be duplicated in these previous studies was the same as that we predict is duplicated in UK48 and UK76; however, at 180 and 300 kbp, our predicted duplications are longer than any of those observed previously. The identification of two additional strains carrying a duplication of the same region suggests that IS-mediated duplication is occurring more frequently in *B. pertussis* than previously believed. Furthermore, the apparent heterogeneity of our UK76 culture suggests that only a portion of UK76 cells may carry the duplication, a phenomenon previously unobserved in any duplication-carrying *B. pertussis* isolate. Finally, the locus of the duplication itself, which contains many motility-related genes, may have interesting implications for an organism traditionally described as non-motile.

Neither our nanopore-only pipeline using Flye nor our hybrid pipeline using Unicycler was able to resolve the duplication correctly, however. The nanopore-only pipeline produced closed contigs for all five strains, seemingly missing the duplication completely, whilst the hybrid pipeline produced a multi-contig assembly for UK48 and the same closed contig as the nanopore-only pipeline for UK76. Our UK48 reads ranged from 73 to 108 575 bp with a mean length of 6243 bp, whilst the UK76 reads ranged from 4 to 70 486 bp with a mean length of 5480 bp; if the key to resolving long repeats is to use reads longer than the longest repeat, we will need high coverage of ultra-long reads in the order of hundreds of thousands of bases to resolve these putative duplications via sequencing [16, 17]. Nanopore sequencing is currently the only sequencing method theoretically capable of producing such long reads; methods to obtain ultra-long reads are under development by the nanopore community, with reports of reads in the order of millions of bases [54].

### Accuracy of long-read sequencing is improving but error estimation is challenging

In addition to our primary aim, we also compared a variety of *de novo* assembly strategies to determine the current optimal pipeline for producing the most accurate genome sequences for *B. pertussis*.

Without a recent, closely related reference sequence, error estimation in *B. pertussis* assemblies is inexact. Comparison with the Tohama I reference sequence will identify basecalling errors which are false positives, having arisen due to natural variation between different strains (that is, true SNPs will be identified as errors). Moreover, the validity of Tohama I as a representative of all *B. pertussis* strains is questionable [55]. The Illumina reads available for four of our sequenced strains (UK36, 38, 39 and 48) showed 98.44 % identity with the Tohama I sequence, suggesting natural genetic variation between Tohama I and these UK strains of around 1.5 %. The false positive rate is thus around 1.5 % when using Tohama I to assess assembly accuracy. On the other hand, comparison with Illumina-only assemblies requires short read data to be available, and assumes the Illumina reads to be close to 100 % accurate, which could be a flawed assumption. The Illumina reads for UK76, for example, had raw identity of only 87.32 % compared to Tohama I. With no distinctive features noted for UK76 in our assembly or in the original comparison of UK epidemic strains [7], it is unlikely that the UK76 genome is truly 11 % less like Tohama I than the other strains sequenced here. It seems more likely that the Illumina reads are inaccurate; if this is the case, our assessments of the accuracy of our UK76 assemblies were skewed. This could explain why our UK76 hybrid assembly had a slightly lower estimated accuracy than the other strains. Compared to Tohama I, our hybrid UK76 assembly showed 98.49 % identity, similar to the identity of our other hybrid assemblies (*n*=5, mean=98.57 %), suggesting that the inaccuracies of the raw Illumina reads do not translate into inaccuracies in the final assembly; only our estimation of accuracy by comparison to the Illumina-only draft is affected. Overall, neither comparison to the Tohama I reference nor comparison to an Illumina-only assembly is ideal for assessing error when working with novel strains, and neither strategy gives us a completely accurate estimate, but using a combination of both comparisons allows a good estimate of assembly error.

Having estimated our hybrid assemblies to be, on average, 99.69 % accurate, we can conclude that roughly 13 000 bases in each 4.1 Mbp draft genome are incorrect. Whilst these incorrectly called bases will not influence comparisons of genome arrangement, residual base errors in draft genome sequences assembled using long reads remain a concern, with the potential to falsely identify SNPs or prevent accurate protein prediction [56]. Incorrect sequencing of homopolymers is a known weakness of many sequencing methods, including nanopore sequencing [17], and our assemblies are no exception. Indeed, a base-level manual comparison of one of our hybrid assemblies with a more accurate Illumina-only draft using progressiveMauve revealed that every difference occurred in a homopolymeric tract, with the hybrid sequence having inserted or deleted bases. Two options for correct SNP identification, therefore, are manual correction of known homopolymeric indels [56], and simply ignoring SNPs which appear to occur in homopolymeric regions. The manual correction option would be time-consuming, whilst the second option could result in false negatives. Nevertheless, until improved pore chemistry or basecalling tools are available which do not produce homopolymeric indels, the use of either option means that SNP identification is still possible, even in assemblies which are less than 100 % accurate.

Correct prediction of proteins appears to be of less concern than SNP identification in our hybrid assemblies: all 40 potential bacterial BUSCOs were present in full for all of our strains, and both Quast and Prokka were able to identify the majority of the Tohama I reference proteins in the same assemblies. In addition, assessment of our UK36 hybrid using Watson’s Ideel pipeline [48] suggested that, although we know some errors remain, they do not substantially inhibit the correct prediction of full-length proteins during annotation. It is here, however, that we can see clearly the benefit of the hybrid assemblies over the nanopore-only assemblies: although the mean accuracy of the nanopore-only assemblies (99.48 %) was only 0.2 % lower than that of the hybrids, none of the nanopore-only assemblies contained full copies of all 40 BUSCOs.

### Does the de Bruijn graph method assemble highly repetitive prokaryotic genomes more accurately than other commonly used methods?

The opinion of the sequencing community has long been that de Bruijn graph assembly is not as effective for error-prone long reads as other *de novo* assembly methods [57, 58]. The tool which consistently produced the most accurate nanopore-only *B. pertussis* assemblies was therefore unexpected: the percentage identity and indel rates of our ABruijn assemblies were better by far than those of the Canu, Miniasm or Unicycler assemblies. The recent version change of ABruijn to Flye seems to have negatively affected these metrics in some of our strains; however, whilst the ABruijn assemblies were better than the Flye assemblies, the Flye assemblies were still better than those produced by other tools. Another recent study, which assembled highly complex and repetitive *Pseudomonas koreensis* genomes using ultra-long nanopore reads, also found Flye to produce the most accurate assemblies [16]. This suggests that the de Bruijn method might be optimal for prokaryotic genomes which contain a high number of repeats.

### Two possible pipelines for *B. pertussis* genome sequence resolution

We have shown here that resolution of five *B. pertussis* genomes per MinION flow cell is possible, whether using long reads alone or in combination with short reads. Sequencing five strains using one flow cell produced a mean yield of over 300× *B. pertussis* genome coverage per strain, which probably exceeds that required to achieve comparable results. A draft produced from using roughly half of our reads (175× coverage) for UK36, pre-corrected and assembled with Flye, had an identity of 99.467 %, whilst the same assembly produced by the full (360× coverage) read set had an identity of 99.474 %. This suggests that twice as many strains could be *de novo* assembled per flow cell without a notable drop in accuracy. Thus, resolution of ten *B. pertussis* genomes per MinION flow cell should be possible.

If short reads are also available, we have shown that hybrid assembly, using pre-correction with Canu followed by Unicycler, remains the most accurate method. Indeed, for now, for full strain characterization (including comparison of genome arrangement, SNP identification and allele-typing), hybrid assemblies are required. For comparison of genome structure and arrangement only (e.g. Fig. 3), however, our nanopore-only pipeline, which uses Canu pre-correction, Flye assembly and post-assembly polishing with Nanopolish, can produce single contig assemblies of adequate accuracy for all but the most unusual *B. pertussis* genomes.

### Continued improvement of long-read data processing tools

Although the pipelines we have defined here produce the most accurate *B. pertussis* genome sequences currently possible, the tools available for the analysis of nanopore sequencing data are continually improving. A recent update to Racon added the ability to polish assemblies with Illumina reads; a brief comparison of this with Pilon, however, showed little improvement to our data, so we did not add short-read Racon polishing to our suite of tests. Alternative basecallers such as Chiron [59] or the currently in-development Guppy, which use entirely new basecalling algorithms, may also offer further accuracy improvements and could be trialled with existing and future *B. pertussis* data sets, particularly if Illumina short reads are not available for hybrid assembly.

We tested the most commonly used *de novo* assembly tools suitable for long reads and, at the time of writing, are not aware of any newly released tools. However, minor (or sometimes major, in the case of ABruijn to Flye) updates are common. New polishing tools are also being developed: ONT’s own Medaka, for example, is claimed to rival Nanopolish in terms of speed and assembly improvement capabilities [60]. In addition, MaSuRCA [61] was not trialled here due to the low Illumina coverage (the manual suggests 50×+ for hybrid assemblies, whereas we had only 37.5× coverage for UK36). Ultimately, for the foreseeable future, no data pipeline including nanopore reads should be set in stone; we will continue to trial new tools and to update our pipeline where appropriate, and would suggest that similar pipeline optimization may be required for each organism to be sequenced.

## Data bibliography

1. Nanopore reads, basecalled with MinKNOW and demultiplexed with Porechop, figshare https://doi.org/10.6084/m9.figshare.6323099 (2018).
2. Nanopore reads, basecalled and demultiplexed with Albacore only, figshare https://doi.org/10.6084/m9.figshare.6302042.v1 (2018).
3. Nanopore reads, basecalled and demultiplexed with Albacore, re-demultiplexed with Porechop (used for final assemblies, includes separate FFPE and non-FFPE reads), figshare https://doi.org/10.6084/m9.figshare.6294791.v2 (2018).
4. Best Nanopore-only assembly per tool tested, figshare https://doi.org/10.6084/m9.figshare.6462767.v1 (2018).
5. Best hybrid assembly per tool tested, figshare https://doi.org/10.6084/m9.figshare.6462773.v2 (2018).
6. Illumina reads, downloaded from SRA and trimmed using Trimmomatic (for hybrid assemblies), figshare https://doi.org/10.6084/m9.figshare.6833492.v1 (2018).
7. ABySS-assembled Illumina-only contigs and Tohama I used for error estimation, and IS element sequences, figshare https://doi.org/10.6084/m9.figshare.6462446.v2 (2018).
8. Final nanopore-only assemblies for all strains, assembled using the pipeline shown in fig. 2, figshare https://doi.org/10.6084/m9.figshare.6670721.v1 (2018).
9. Final hybrid assemblies for all strains, assembled using the pipeline shown in fig 2. (annotations available from GenBank), figshare https://doi.org/10.6084/m9.figshare.6670454.v2 (2018).

## Supplementary Data

Supplementary File 1

## References

[1] Burns DL, Meade BD, Messionnier NE (2014). Pertussis resurgence: perspectives from the Working Group Meeting on pertussis on the causes, possible paths forward, and gaps in our knowledge. J Infect Dis.

[2] Jakinovich A, Sood SK (2014). Pertussis: still a cause of death, seven decades into vaccination. Curr Opin Pediatr.

[3] Sealey K (2015). Is the Circulating Uk Bordetella Pertussis Population Evolving to Evade Vaccine-Induced Immunity?.

[4] Clark TA (2014). Changing pertussis epidemiology: everything old is new again. J Infect Dis.

[5] Ausiello CM, Cassone A (2014). Acellular pertussis vaccines and pertussis resurgence: revise or replace?. MBio.

[6] Bart MJ, Harris SR, Advani A, Arakawa Y, Bottero D (2014). Global population structure and evolution of *Bordetella pertussis* and their relationship with vaccination. MBio.

[7] Sealey KL, Harris SR, Fry NK, Hurst LD, Gorringe AR (2015). Genomic analysis of isolates from the United Kingdom 2012 pertussis outbreak reveals that vaccine antigen genes are unusually fast evolving. J Infect Dis.

[8] Bowden KE, Williams MM, Cassiday PK, Milton A, Pawloski L (2014). Molecular epidemiology of the pertussis epidemic in Washington State in 2012. J Clin Microbiol.

[9] Octavia S, Sintchenko V, Gilbert GL, Lawrence A, Keil AD (2012). Newly emerging clones of *Bordetella* pertussis carrying prn2 and ptxP3 alleles implicated in Australian pertussis epidemic in 2008– 2010. J Infect Dis.

[10] Lam C, Octavia S, Ricafort L, Sintchenko V, Gilbert GL (2014). Rapid increase in pertactin-deficient *Bordetella pertussis* isolates, Australia. Emerg Infect Dis.

[11] Chin CS, Alexander DH, Marks P, Klammer AA, Drake J (2013). Nonhybrid, finished microbial genome assemblies from long-read SMRT sequencing data. Nat Methods.

[12] Conlan S, Thomas PJ, Deming C, Park M, Lau AF (2014). Single-molecule sequencing to track plasmid diversity of hospital-associated carbapenemase-producing Enterobacteriaceae. Sci Transl Med.

[13] Koren S, Phillippy AM (2015). One chromosome, one contig: complete microbial genomes from long-read sequencing and assembly. Curr Opin Microbiol.

[14] Loman NJ, Quick J, Simpson JT (2015). A complete bacterial genome assembled de novo using only nanopore sequencing data. Nat Methods.

[15] Wick RR, Judd LM, Gorrie CL, Holt KE (2017). Completing bacterial genome assemblies with multiplex MinION sequencing. Microb Genom.

[16] Schmid M, Frei D, Patrignani A, Schlapbach R, Frey JE (2018). Pushing the limits of *de novo* genome assembly for complex prokaryotic genomes harboring very long, near identical repeats. Nucleic Acids Res.

[17] Jain M, Koren S, Miga KH, Quick J, Rand AC (2018). Nanopore sequencing and assembly of a human genome with ultra-long reads. Nat Biotechnol.

[18] Jain M, Olsen HE, Turner DJ, Stoddart D, Bulazel KV (2018). Linear assembly of a human centromere on the Y chromosome. Nat Biotechnol.

[19] Bentley DR, Balasubramanian S, Swerdlow HP, Smith GP, Milton J (2008). Accurate whole human genome sequencing using reversible terminator chemistry. Nature.

[20] Siguier P, Gourbeyre E, Chandler M (2014). Bacterial insertion sequences: their genomic impact and diversity. FEMS Microbiol Rev.

[21] Bowden KE, Weigand MR, Peng Y, Cassiday PK, Sammons S (2016). Genome structural diversity among 31 *Bordetella pertussis* isolates from two recent U.S. whooping cough statewide epidemics. mSphere.

[22] Weigand MR, Peng Y, Loparev V, Batra D, Bowden KE (2017). The history of *Bordetella pertussis* genome evolution includes structural rearrangement. J Bacteriol.

[23] Parkhill J, Sebaihia M, Preston A, Murphy LD, Thomson N (2003). Comparative analysis of the genome sequences of *Bordetella pertussis*, *Bordetella parapertussis* and *Bordetella bronchiseptica*. Nat Genet.

[24] Preston A, Parkhill J, Maskell DJ (2004). The bordetellae: lessons from genomics. Nat Rev Microbiol.

[25] Heikkinen E, Kallonen T, Saarinen L, Sara R, King AJ (2007). Comparative genomics of *Bordetella pertussis* reveals progressive gene loss in Finnish strains. PLoS One.

[26] Caro V, Hot D, Guigon G, Hubans C, Arrivé M (2006). Temporal analysis of French *Bordetella pertussis* isolates by comparative whole-genome hybridization. Microbes Infect.

[27] Bayliss SC, Hunt VL, Yokoyama M, Thorpe HA, Feil EJ (2017). The use of Oxford Nanopore native barcoding for complete genome assembly. Gigascience.

[28] Ton KNT, Cree SL, Gronert-Sum SJ, Merriman TR, Stamp LK (2017). Multiplexed nanopore sequencing of HLA-B locus in Māori and Polynesian samples. bioRxiv.

[29] Pomerantz A, Penafiel N, Arteaga A, Bustamante L, Pichardo F (2017). Real-time DNA barcoding in a remote rainforest using nanopore sequencing. bioRxiv.

[30] Quick J, Loman NJ, Duraffour S, Simpson JT, Severi E (2016). Real-time, portable genome sequencing for Ebola surveillance. Nature.

[31] Edwards A, Debbonaire AR, Sattler B, Mur LA, Hodson AJ (2016). Extreme metagenomics using nanopore DNA sequencing: a field report from Svalbard 78 N. bioRxiv.

[32] Connor TR, Loman NJ, Thompson S, Smith A, Southgate J (2016). CLIMB (the Cloud Infrastructure for Microbial Bioinformatics): an online resource for the medical microbiology community. Microb Genom.

[33] Simpson JT, Wong K, Jackman SD, Schein JE, Jones SJ (2009). ABySS: a parallel assembler for short read sequence data. Genome Res.

[34] Bolger AM, Lohse M, Usadel B (2014). Trimmomatic: a flexible trimmer for Illumina sequence data. Bioinformatics.

[35] Gummy-Bear (2014). Calculate length of all sequences in an multi-fasta file. https://bioexpressblog.wordpress.com/2014/04/15/calculate-length-of-all-sequences-in-an-multi-fasta-file/.

[36] Li H (2013). Aligning sequence reads, clone sequences and assembly contigs with BWA-MEM. arXiv.

[37] Li H, Handsaker B, Wysoker A, Fennell T, Ruan J (2009). The Sequence Alignment/Map format and SAMtools. Bioinformatics.

[38] Lin Y, Yuan J, Kolmogorov M, Shen MW, Chaisson M (2016). Assembly of long error-prone reads using de Bruijn graphs. Proc Natl Acad Sci USA.

[39] Koren S, Walenz BP, Berlin K, Miller JR, Bergman NH (2017). Canu: scalable and accurate long-read assembly via adaptive *k*-mer weighting and repeat separation. Genome Res.

[40] Li H (2016). Minimap and miniasm: fast mapping and de novo assembly for noisy long sequences. Bioinformatics.

[41] Wick RR, Judd LM, Gorrie CL, Holt KE (2017). Unicycler: Resolving bacterial genome assemblies from short and long sequencing reads. PLoS Comput Biol.

[42] Vaser R, Sović I, Nagarajan N, Šikić M (2017). Fast and accurate *de novo* genome assembly from long uncorrected reads. Genome Res.

[43] Walker BJ, Abeel T, Shea T, Priest M, Abouelliel A (2014). Pilon: an integrated tool for comprehensive microbial variant detection and genome assembly improvement. PLoS One.

[44] Bankevich A, Nurk S, Antipov D, Gurevich AA, Dvorkin M (2012). SPAdes: a new genome assembly algorithm and its applications to single-cell sequencing. J Comput Biol.

[45] Wick RR, Judd LM, Holt KE (2018). Comparison of Oxford Nanopore basecalling tools. https://github.com/rrwick/Basecalling-comparison.

[46] Gurevich A, Saveliev V, Vyahhi N, Tesler G (2013). QUAST: quality assessment tool for genome assemblies. Bioinformatics.

[47] Simão FA, Waterhouse RM, Ioannidis P, Kriventseva EV, Zdobnov EM (2015). BUSCO: assessing genome assembly and annotation completeness with single-copy orthologs. Bioinformatics.

[48] Watson M (2018). A simple test for uncorrected insertions and deletions (indels) in bacterial genomes. Opiniomics.

[49] Seemann T (2014). Prokka: rapid prokaryotic genome annotation. Bioinformatics.

[50] Darling AE, Mau B, Perna NT (2010). progressiveMauve: multiple genome alignment with gene gain, loss and rearrangement. PLoS One.

[51] Bairoch A, Apweiler R (2000). The SWISS-PROT protein sequence database and its supplement TrEMBL in 2000. Nucleic Acids Res.

[52] Weigand MR, Pawloski LC, Peng Y, Ju H, Burroughs M (2018). Screening and genomic characterization of filamentous hemagglutinin-deficient *Bordetella pertussi*s. Infect Immun.

[53] Weigand MR, Peng Y, Loparev V, Johnson T, Juieng P (2016). Complete genome sequences of four *Bordetella pertussis* vaccine reference strains from Serum Institute of India. Genome Announc.

[54] Payne A, Holmes N, Rakyan V, Loose M (2018). Whale watching with BulkVis: a graphical viewer for Oxford Nanopore bulk fast5 files. bioRxiv.

[55] Caro V, Bouchez V, Guiso N (2008). Is the sequenced *Bordetella pertussis* strain Tohama I representative of the species?. J Clin Microbiol.

[56] Watson M (2018). Mind the gaps - ignoring errors in long read assemblies critically affects protein prediction. bioRXiv.

[57] Lu H, Giordano F, Ning Z (2016). Oxford Nanopore MinION sequencing and genome assembly. Genomics Proteomics Bioinformatics.

[58] Pop M (2009). Genome assembly reborn: recent computational challenges. Brief Bioinform.

[59] Teng H, Cao MD, Hall MB, Duarte T, Wang S (2018). Chiron: translating nanopore raw signal directly into nucleotide sequence using deep learning. Gigascience.

[60] Nanoporetech (2018). Clive G Brown: CTO plenary from London Calling. https://nanoporetech.com/about-us/news/clive-g-brown-cto-plenary-london-calling?keys=MinION&page=28.

[61] Zimin AV, Puiu D, Luo MC, Zhu T, Koren S (2017). Hybrid assembly of the large and highly repetitive genome of *Aegilops tauschii*, a progenitor of bread wheat, with the MaSuRCA mega-reads algorithm. Genome Res.

